# Glia Maturation Factor and Mitochondrial Uncoupling Proteins 2 and 4 Expression in the Temporal Cortex of Alzheimer’s Disease Brain

**DOI:** 10.3389/fnagi.2017.00150

**Published:** 2017-05-18

**Authors:** Ramasamy Thangavel, Duraisamy Kempuraj, Smita Zaheer, Sudhanshu Raikwar, Mohammad E. Ahmed, Govindhasamy Pushpavathi Selvakumar, Shankar S. Iyer, Asgar Zaheer

**Affiliations:** ^1^Department of Neurology, Center for Translational Neuroscience, School of Medicine, University of MissouriColumbia, MO, United States; ^2^Research Services, Harry S. Truman Memorial Veterans HospitalColumbia, MO, United States

**Keywords:** Alzheimer’s disease, glia maturation factor, mitochondrial uncoupling protein, inducible nitric oxide, NF-κB, para hippocampal gyrus, UCP2, UCP4

## Abstract

Alzheimer’s disease (AD) is characterized by the presence of neuropathological lesions containing amyloid plaques (APs) and neurofibrillary tangles (NFTs). AD is associated with mitochondrial dysfunctions, neuroinflammation and neurodegeneration in the brain. We have previously demonstrated enhanced expression of the proinflammatory protein glia maturation factor (GMF) in glial cells near APs and NFTs in the AD brains. Parahippocampal gyrus consisting of entorhinal and perirhinal subdivisions of temporal cortex is the first brain region affected during AD pathogenesis. Current paradigm implicates oxidative stress-mediated neuronal damage contributing to the early pathology in AD with mitochondrial membrane potential regulating reactive oxygen species (ROS) production. The inner mitochondrial membrane anion transporters called the uncoupling proteins (UCPs), function as regulators of cellular homeostasis by mitigating oxidative stress. In the present study, we have analyzed the expression of GMF and mitochondrial UCP2 and UCP4 in the parahippocampal gyrus of AD and non-AD brains by immunostaining techniques. APs were detected by thioflavin-S fluorescence staining or immunohistochemistry (IHC) with 6E10 antibody. Our current results suggest that upregulation of GMF expression is associated with down-regulation of UCP2 as well as UCP4 in the parahippocampal gyrus of AD brains as compared to non-AD brains. Further, GMF expression is associated with up-regulation of inducible nitric oxide synthase (iNOS), the enzyme that induces the production of nitric oxide (NO), as well as nuclear factor kB p65 (NF-κB p65) expression. Also, GMF appeared to localize to the mitochondria in AD brains. Based on our current observations, we propose that enhanced expression of GMF down-regulates mitochondrial UCP2 and UCP4 thereby exacerbating AD pathophysiology and this effect is potentially mediated by iNOS and NF-κB. Thus, GMF functions as an activator protein that interferes with the cytoprotective mechanisms in AD brains.

## Introduction

Alzheimer’s disease (AD) is a devastating progressive neurodegenerative disease affecting 5.5 million Americans and about 35 million people worldwide. If the present trends continue, by 2050 there will be an estimated 14 million AD patients in US alone. AD is characterized by the presence of amyloid plaques (APs) and neurofibrillary tangles (NFTs) containing hyper phosphorylated Tau and is associated with significantly increased numbers of activated glia and severe neuronal loss in the AD brains (Pekny and Nilsson, 2005; Streit et al., 2009; Thangavel et al., 2012). NFTs and APs are first detectable in the aged as well as in AD brains in the parahippocampal gyrus consisting the entorhinal cortex is the area where NFTs and APs are first detectable in the aged as well as in AD brains (Bevilaqua et al., 2008). The involvement of parahippocampal gyrus in AD pathophysiology is already reported (Van Hoesen et al., 2000). Entorhinal cortex is the earliest as well as the most vulnerable and heavily affected brain region in AD (Hof et al., 2003; Porchet et al., 2003). Inflammation is the hallmark of chronic neurodegenerative diseases, and transcription factor nuclear factor-kB (NF-κB) is considered an important regulator of these inflammatory processes (Granic et al., 2009). Glia maturation factor (GMF) is an inflammatory protein first discovered, purified and sequenced in our laboratory (Lim et al., 1989, 1990; Kaplan et al., 1991). We have previously shown that overexpression of GMF induces neuroinflammation leading to the death of neurons in the neurodegenerative diseases (Zaheer et al., 2007a, 2008). Our previous studies have also shown that GMF is expressed at the vicinity of APs and NFTs in temporal cortex (Zaheer et al., 2011; Thangavel et al., 2012), entorhinal cortex and hippocampus in AD brains (Stolmeier et al., 2013; Thangavel et al., 2013).

Mitochondrial dysfunction plays a crucial role in the development and progression of the neurodegenerative diseases such as AD (Reddy and Reddy, 2011) and oxidative stress is one of the earliest and significant events in AD pathogenesis (Viña et al., 2007; Kamat et al., 2014). Mitochondria are the main sites for reactive oxygen species (ROS) production. Increased expression of inducible nitric oxide synthase (iNOS) around the plaques has been shown to contribute to the oxidative stress in AD brains (Wong et al., 2001). Uncoupling proteins (UCPs) are inner mitochondrial proteins that protect neurons by reducing the production of free radicals (Lu et al., 2014). The genetic variability of the UCPs in humans has been shown to be associated with longevity by modulating cellular energy level through control of oxidative stress (Rose et al., 2011). The UCPs are endogenous neuroprotective agents and the induction of UCPs in the specific brain region has been suggested to protect neurons from oxidative stress-mediated tissue damage (Conti et al., 2005). Nitric oxide (NO) pathway with iNOS is involved in the UCP expression dynamics in the disease conditions (Litvinova et al., 2015). Previous study has shown that inflammatory cytokines such as tumor necrosis factor-alpha (TNF-α) decreases expression of UCPs through NOS pathway (Merial et al., 2000). Cumulative oxidative stress through enhanced ROS and reactive nitrogen species (RNS) in conjunction with UCPs potentially triggers the neuronal loss. In this context, the relevance of GMF that regulates both arms of this disorder becomes significant. Hence, the present study was carried out to demonstrate the expression of GMF, UCP2, UCP4, iNOS and NF-kB in the parahippocampal gyrus of AD brains using immunohistochemistry (IHC) and immunofluorescence staining procedures.

## Materials and Methods

### Antibodies and Reagents

Rabbit GMF polyclonal antibody and mouse GMF monoclonal antibodies were purchased from Protein Tech (Chicago, IL, USA). The vendors for other antibodies are listed within parentheses. Anti-UCP2 polyclonal antibody (Calbiochem Millipore, San Diego, CA, USA), Rabbit polyclonal UCP4 antibody (Novus Biologicals, Littleton, CO, USA), anti-rabbit iNOS and rabbit polyclonal to NF-κB p65 (Abcam, Cambridge, MA, USA) were obtained. Vectastain avidin biotin peroxidase complex (ABC) reagents and kits (peroxidase mouse IgG/ peroxidase rabbit IgG) and Impact 3,3’-diaminobenzidine (DAB) peroxidase substrate kits were from Vector Labs (Burlingame, CA, USA). Additional reagents include phosphate buffered saline (PBS; GIBCO, Life Technologies, Grand Island, NY, USA), Thioflavin-S (Sigma, St Louis, MO, USA). Permount (Fisher Scientific (Pittsburgh, PA, USA) and Fluorogel (EM Sciences, Hatfield, PA, USA).

### Human AD Brain Samples

Temporal lobes from human postmortem rapid brains of AD patients (*n* = 10) and age matched non-AD control subjects (*n* = 10) were obtained through the University of Iowa Deeded body program and fixed in 4% paraformaldehyde. They were cut into 40 μm thick sections on a sledge freezing microtome and the sections were collected in PBS and stored in cryo storage solution (glycerol 30 ml, ethylene glycol 30 ml, 40 ml 0.1 M PBS) until used for immunostaining. This study was approved by the University of Missouri Institutional Review Board (IRB #2008067; Exempt Application 224561), Columbia, MO, USA. This study was conducted under standard ethical procedures. All the appropriate personal protection safety procedures were followed to handle the human samples.

### IHC for UCP2 or UCP4 with Thioflavin-S Double Staining

Free-floating sections of parahippocampal gyrus were treated with 0.3% hydrogen peroxide (in PBS) solution for 20 min at room temperature (RT). After washing in PBS, the sections were incubated in blocking buffer (5% normal goat serum, 3% bovine serum albumin (BSA) and 0.1% Triton-X in PBS) for 1 h at RT. Then the sections were incubated overnight at 4°C with either anti-UCP2 (1:500 dilutions) or anti-UCP4 (1:500 dilutions). Next day, the sections were washed in PBS and incubated for 1–2 h with appropriate biotinylated goat anti-mouse IgG or goat anti-rabbit IgG secondary antibody. The sections were rinsed again in PBS and developed with an ABC standard staining kit solution diluted in PBS for 1 h. After washing, sections were incubated with Impact DAB peroxidase solution for 5 min and counterstained with thioflavin-S to show the association between UCPs and NFTs or APs in AD and non-AD brains as described earlier (Thangavel et al., 2013). The sections were rinsed with distilled water, mounted on slides and dried. Slides were then dehydrated, cleared in xylene and cover slipped with Permount.

### Double Immunofluorescence for GMF with UCP2 or UCP4

Free floating sections of parahippocampal gyrus from AD and non-AD brains were incubated with a mixture of GMF monoclonal antibody and polyclonal UCP2 or UCP4 antibodies overnight at 4°C (Thangavel et al., 2013). Then the sections were incubated for 1 h at RT with the appropriate secondary antibodies. Monoclonal GMF was visualized with goat anti-mouse IgG conjugated with green fluorescent dye Alexa Fluor 488. Polyclonal UCP2 and UCP4 were visualized with goat anti-rabbit IgG conjugated with red fluorescent dye Alexa Fluor 568. Then the sections were rinsed and cover-slipped with Fluorogel and observed under a Nikon (DIAPHOT) microscope (Garden City, NY, USA).

Additionally, imaging of human AD brain sections was conducted on a Leica TCP SP8 laser scanning confocal microscope with a 405-nm diode laser and tunable super continuum white light laser using 63× oil immersion objective. Briefly, the brain sections were incubated with GMF monoclonal antibody and UCP2 polyclonal antibody. UCP2 (green) was visualized with goat anti-rabbit IgG conjugated with green fluorescent dye Alexa Fluor 488 and GMF (red) was visualized with goat anti-mouse IgG conjugated with green fluorescent dye Alexa Fluor 568. Double immunofluorescence labeling of AD brain sections were performed with GMF monoclonal antibody and UCP4 polyclonal antibody. UCP4 (green) was visualized with goat anti-rabbit IgG conjugated with green fluorescent dye Alexa Fluor 488 and GMF (red) was visualized with goat anti-mouse IgG conjugated with green fluorescent dye Alexa Fluor 568. The following excitation/emission band-pass wavelengths were used: 405/420–480 nm (DAPI), 495/505–550 nm (Alexa Fluor 488) and 570/580–630 nm (Alexa Fluor 568). Confocal images of double immunofluorescence labeling for GMF and UCP2, GMF and UCP4, GMF and voltage-dependent anion-selective channel 1 (VDAC1; ThermoFisher Scientific, Rockford, IL, USA), 6E10 and fatty acid synthase (FASN) antibody (Cambridge, MA, USA), NF-κB and DAPI in the brain sections of AD were acquired.

### Quantitation of GMF, UCP2 and UCP4 Positive Cells and Statistical Analysis

UCP2 and UCP4-positive cells were counted in the double stained slides. GMF and UCP2-positive cells or GMF and UCP4-positive cells were counted in the double immunofluorescence slides (Thangavel et al., 2013; Xiong et al., 2014). To quantitate the staining, we counted UCP2, UCP4 or GMF- positive cells under 400× total magnifications in 5 different fields in AD and non-AD brains and then averaged. The data were presented as the number of GMF, UCP2 and UCP4-positive cells/95 mm^2^. The data were analyzed by unpaired *t*-test or nonparametric Mann-Whitney test. A *p*-value of <0.05 was considered statistically significant.

### Double Immunofluorescence for GMF with iNOS or NF-κB

Free floating sections of parahippocampal gyrus were incubated with a mixture of GMF monoclonal antibody and polyclonal iNOS or polyclonal NF-κB p65 antibodies for overnight at 4°C (Thangavel et al., 2013). Then the sections were incubated for 1 h at RT with the appropriate secondary antibodies. GMF was visualized following labeling with goat anti-mouse IgG conjugated with green fluorescent dye Alexa Fluor 488, and iNOS or NF-κB were visualized post labeling with goat anti-rabbit IgG conjugated with red fluorescent dye Alexa Fluor 568. The sections were rinsed and cover-slipped with Fluorogel and the images were acquired on a Nikon (DIAPHOT) fluorescence microscope.

### Double IHC for GMF with iNOS or NF-κB

The parahippocampal gyrus sections were double immunostained to detect GMF (1:100 dilutions) with iNOS (1:100) or NF-κB p65 (1:100 dilutions). For two-color double labeling, the sections were first incubated with GMF monoclonal antibody (1:100 dilutions) overnight at 4°C then incubated with DAB substrate solution which produces brown color. Subsequently, these sections were further incubated with iNOS or NF-κB overnight at 4°C. Then the sections were incubated with SG substrate (Vector) solution as second chromogen, which produces blue color. The sections were then mounted on slides, dried, dehydrated, cleared and cover-slipped using Permount.

### Double IHC for APs (6E10 Antibody) with Anti-iNOS or NF-κB

We then performed co-localization of the APs (6E10 antibody) with iNOS or NF-κB in the parahippocampal gyrus sections of the AD brains. IHC with 6E10 was performed first using the procedure described above. Briefly, after the visualization of immunoreactivity with DAB (brown color), the sections were rinsed in PBS and blocked for 1 h at RT and the slides were incubated with primary antibodies against GMF, iNOS or NF-κB at 4°C for overnight. After washing with PBS, the sections were incubated with ABC (diluted 1:50) solution for 1 h at RT. The second immunoperoxidase reaction was developed with SG substrate solution (Vector, blue color). Sections were then mounted on slides, dried, dehydrated, cleared and cover-slipped with Permount.

## Results

### IHC Detection of Down-Regulation of UCP2 and UCP4 in AD Brains

**Figure 1:**
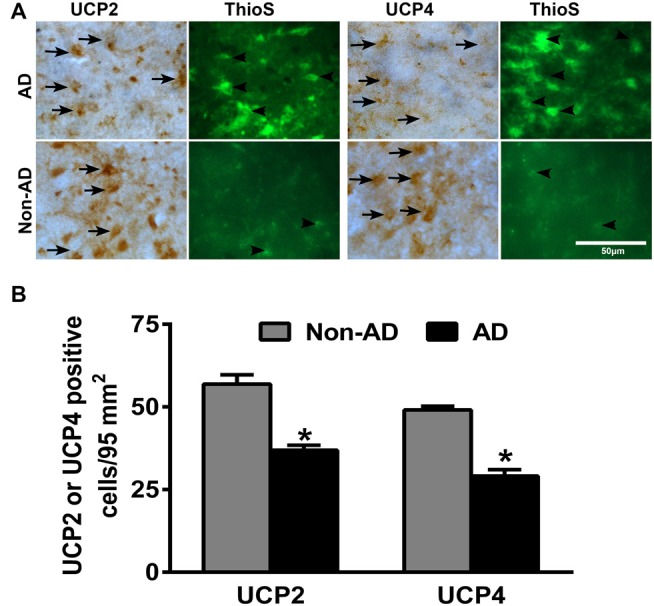
**Immunohistochemistry (IHC) detection of uncoupling proteins 2 (UCP2) or UCP4 with amyloid plaques (APs) and neurofibrillary tangles (NFTs) in Alzheimer’s disease (AD) and non-AD brains. (A)** Representative photomicrographs show UCP2 or UCP4 IHC staining followed by thioflavin-S fluorescence staining in the parahippocampal gyrus of AD (*n* = 10) and non-AD brains (*n* = 10). Both UCP2 and UCP4 expression (brown color, arrows) were decreased in AD brains when compared to non-AD brains. Thioflavin-S staining (green color, arrowheads) showed APs and NFTs in the AD brain (magnifications = 400×). **(B)** We also counted UCP2 and UCP4-positive cells in AD (*n* = 10) and non-AD (*n* = 10) brains using the IHC slides. The counting was performed under the microscope using high magnification objectives at five different fields in each section and then averaged. The data were presented as mean ± SEM of the number of UCP2 or UCP4-positive cells/95 mm^2^, *p < 0.05, *t* test.

To investigate whether there is differential expression of UCP2 and UCP4 in the AD and non-AD brain sections, we performed IHC for UCP2 and UCP4 expression in the parahippocampal gyrus of AD and non-AD brains. Parahippocampal gyrus showed decrease in the expression of both UCP2 as well as UCP4 in the AD brain when compared to non-AD control brains (brown color, arrows; Figure 1A). After the UCP staining, the same sections were also stained for Thioflavin-S to detect APs and NFTs in the brain. Thioflavin-S staining (green color) shows the presence of APs and NFTs (arrowheads) in AD brain (Figure 1A). Though number of APs increased in AD brains compared to non-AD brains, we did not see any significant association of UCPs expression with APs in AD brains. To quantitate UCPs expression, the numbers of UCP2 or UCP4-positive cells were counted in the IHC slides of AD and non-AD brains (Figure 1B). Expression of both UCP2 as well as UCP4 was significantly decreased (*p* < 0.05) in AD brain sections as compared to non-AD brain sections.

### Double Immunofluorescence Detection of GMF with UCP2 or UCP4 in AD Brains

**Figure 2:**
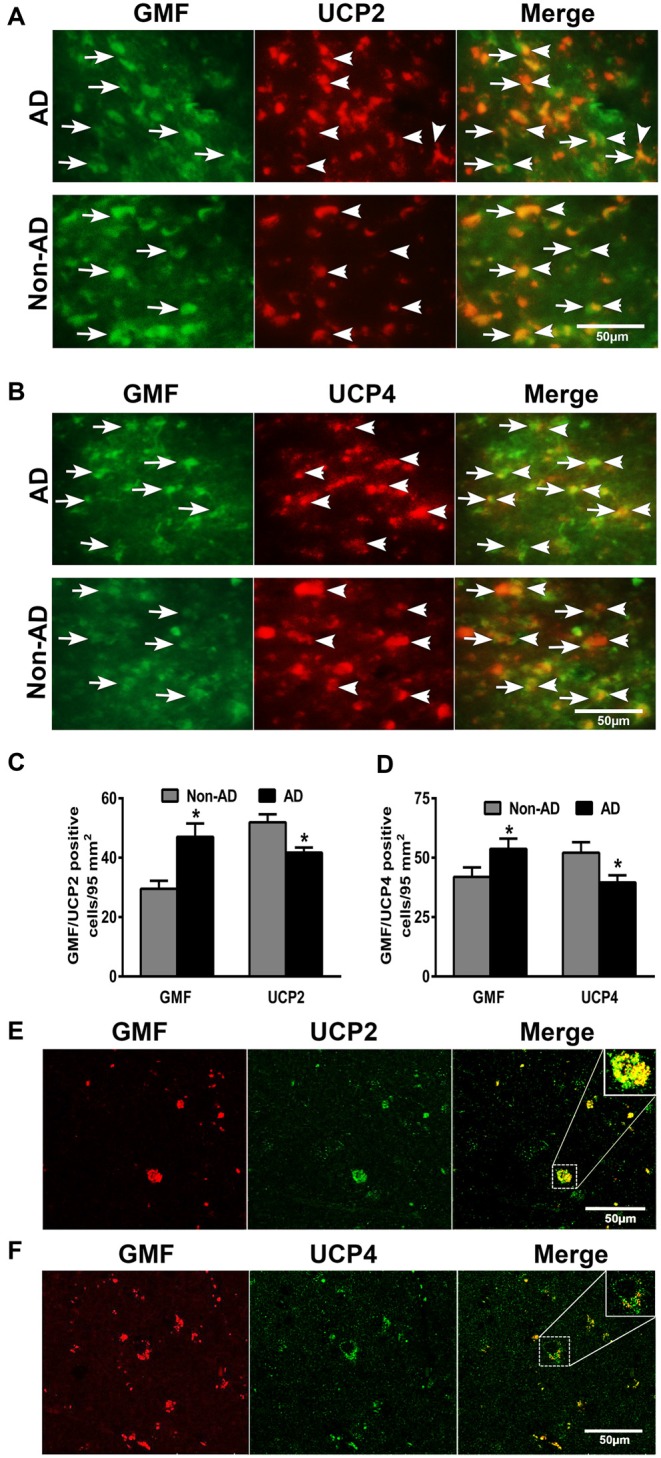
**Double immunofluorescence detection of glia maturation factor (GMF) with UCP2 or UCP4 in parahippocampal gyrus of AD brain and compared with non-AD brain (*n* = 7–10).** GMF monoclonal antibody and UCP2 or UCP4 polyclonal antibodies were mixed and the sections were incubated with this mixture. GMF was visualized using goat anti-mouse IgG conjugated with green fluorescent dye Alexa Fluor 488. **(A)** UCP2 and **(B)** UCP4 proteins were visualized using goat anti-rabbit IgG conjugated with red fluorescent dye Alexa Fluor 568. UCP2 and UCP4 expressions are reduced in AD brains when compare to non-AD brains. We have observed increased expression of GMF (green color, arrows) in AD brain when compare to non-AD brain. UCP2 and UCP4 expressions are observed at the same location where GMF is expressed (magnification = 400×). To quantitate the immunostaining, we counted **(C)** GMF and UCP2 or **(D)** GMF and UCP4-positive cells under the microscope at five different fields in each section and then averaged (*n* = 7–10). The data were presented as mean ± SEM of the number of GMF or UCP2 or UCP4-positive cells/95 mm^2^, *p < 0.05, *t* test. **(E,F)** Confocal microscopic double immunofluorescence labeling of AD brain sections. Sections were incubated with GMF monoclonal antibody and UCP2/UCP4 polyclonal antibodies. UCP2/UCP4 (green) were visualized with goat anti-rabbit IgG conjugated with green fluorescent dye Alexa Fluor 488. GMF (red) labeling visualized with goat anti-mouse IgG conjugated with green fluorescent dye Alexa Fluor 568. Boxed area show colocalization of GMF and UCP2 or UCP4 in the merged image (magnification 63× oil immersion objective).

**Figure 3:**
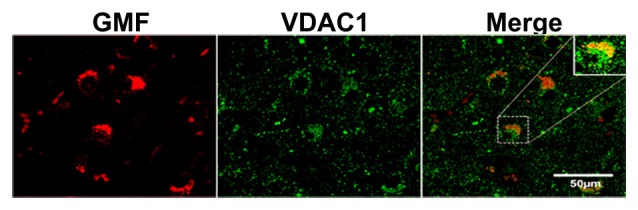
**Confocal microscopy image of double immunofluorescence labeling of GMF (red) and voltagedependent anion-selective channel 1 (VDAC1; green) in the brain section of AD.** Merged image showed the colocalization of GMF and VDAC1. Boxed area show the enlarged view of co-localization of GMF and VDAC1 (magnification 63× oil immersion objective).

To address the question whether GMF interacts with UCP2 and UCP4 we performed colocalization studies. By double immunofluorescence staining procedures, we observed increased expression of GMF (green color) in parahippocampal gyrus of AD brain when compared to non-AD brain (Figure 2). The same sections were stained for both UCP2 (red color) or UCP4 (red color, arrowheads) after GMF staining in AD and non-AD brains. UCP2 (Figure 2A) and UCP4 (Figure 2B) are less abundant in the AD brain when compared to non-AD brains by double immunofluorescence staining similar to IHC results as shown in Figure 1A. Dual immunofluorescence revealed colocalization of UCP2 and UCP4 expressions with GMF (Figure 2). To quantitate the GMF and UCPs expression, the number of GMF and UCP2-positive cells (Figure 2C) or GMF and UCP4 (Figure 2D) -positive cells were counted in AD and non-AD brain sections using immunofluorescence slides. Both UCP2 and UCP4 were significantly decreased (*p* < 0.05) in AD brains when compared to non-AD brains. Results show increased expression of GMF in AD brains when compared to non-AD brains (Figures 2A,B). GMF upregulation was associated with downregulation of UCP2 and UCP4. It is clear from the confocal data (Figures 2E,F) that UCP2 and UCP4s colocalized with GMF. In addition, it is clear that in AD brains, GMF localizes to the mitochondria as shown by colocalization of GMF with the mitochondrial marker-VDAC1 (Figure 3).

### Double Immunofluorescence and Double IHC Detection of GMF Upregulation Associated with Increased iNOS or NF-κB Levels in AD Brains

**Figure 4:**
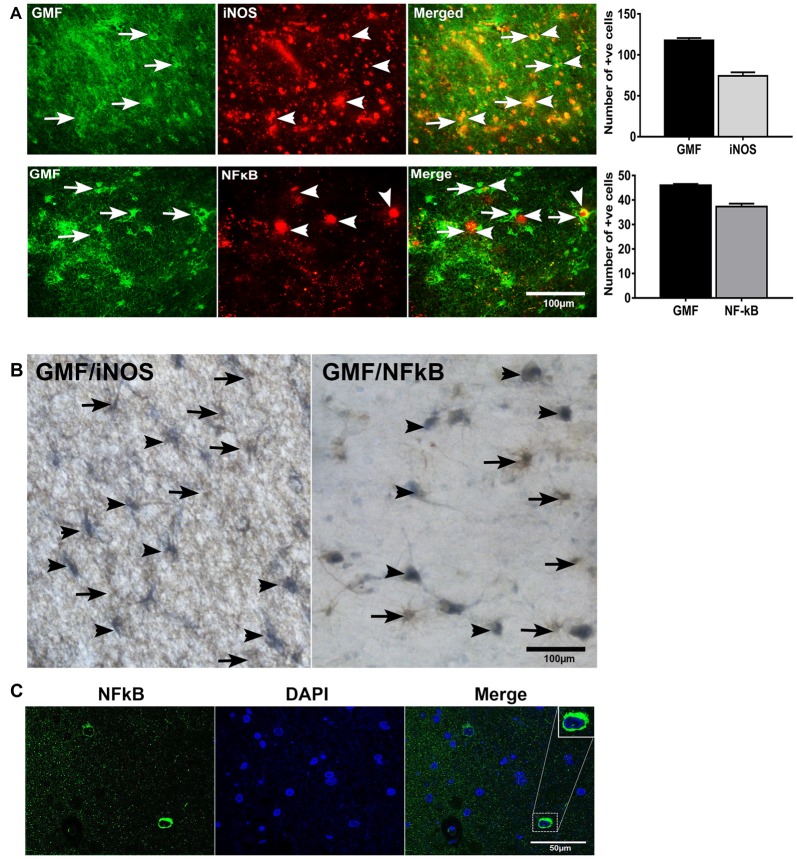
**Double immunofluorescence and double IHC detection of GMF and inducible nitric oxide synthase (iNOS) or GMF and nuclear factor kB p65 (NF-κB p65) in AD brains. (A)** Parahippocampal gyrus sections from AD patient brain were incubated with a mixture of GMF monoclonal antibody and polyclonal iNOS or NF-κB antibodies. GMF was visualized with goat anti-mouse IgG conjugated with green fluorescent dye Alexa Fluor 488, and iNOS or NF-κB were visualized with goat anti-rabbit IgG conjugated with red fluorescent dye Alexa Fluor 568 to iNOS or NF-κB. **(A)** iNOS (red color, arrowheads) and NF-κB (red color, arrowheads) were localized at the vicinity of GMF (green color, arrows) expression. **(A)** Merged image show the co-localization of GMF with iNOS or NF-κB (magnification = 200×). **(B)** We have also co-localized GMF with iNOS and GMF with NF-κB by double IHC staining in AD brains (*n* = 3). The brain sections were first incubated with GMF monoclonal antibody and 3,3’-diaminobenzidine (DAB) substrate solution (brown color) followed by incubation for iNOS or NF-κB detection. Then the sections were incubated with Vector SG substrate solution (blue color). Double IHC staining also revealed the co-localization of GMF (brown color, arrows) with iNOS (blue color, arrowheads) or GMF with NF-κB (blue color, arrowhead). Glial cells show the expression of GMF as well as iNOS or NF-κB (magnification = 400×). Positive cells were counted from the images and presented in bar graphs. **(C)** NF-κB immunofluorescence labeling with DAPI counter staining showing NF-κB cytoplasmic expression. Boxed area show enlarged view of NF-κB and DAPI co-localization (magnification 63× oil immersion objective).

To investigate whether or not GMF interacts with iNOS or NF-κB, first, we performed double immunofluorescence staining to detect GMF with iNOS or GMF with NF-κB in the parahippocampal gyrus of AD brains. Our results show that iNOS (red color, arrowheads upper panel) or NF-κB (red color, arrowheads lower panel) are localized in the vicinity where of GMF is expressed (green color, arrows; Figure 4A). Merged image show the co-localization of GMF with iNOS or NF-κB (Figure 4A). Following immunofluorescence staining for GMF, iNOS and NF-κB, we have also performed non-fluorescence IHC staining to co-localize GMF with iNOS, as well as GMF with NF-κB (Figure 4B). Our double IHC staining also revealed the co-localization of GMF (brown color, arrows) with iNOS (blue color, arrowheads left panel) or GMF with NF-kB (blue color, arrowheads right panel) in the parahippocampal gyrus of AD brains (Figure 4B). Glial cells show the expression of GMF as well as iNOS and NF-κB indicating the involvement of iNOS and NF-κB in GMF-upregulation and release similar to the role of NF-κB in proinflammatory cytokine release during an inflammatory response. Positive cells were counted from these images and shown in bar graphs (Figure 4A). NF-κB immunofluorescence with DAPI nuclear counterstaining showed the cytoplasmic location in the AD brain (Figure 4C).

### Double IHC Detection of APs with iNOS or NF-κB in AD Brains

**Figure 5:**
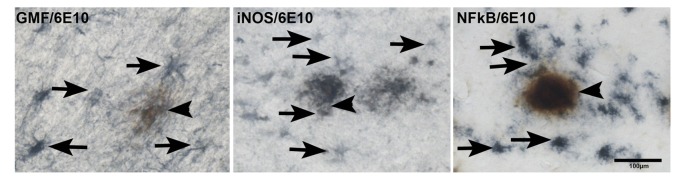
**Double IHC detection of APs with GMF or iNOS or NF-κB in parahippocampal gyrus of AD brains (*n* = 3).** We have performed co-localization of APs (6E10 antibody) with GMF or iNOS or NF-κB subunit p65. We have first performed IHC for APs with 6E10 antibody (brown color, arrowheads). After the visualization of immunoreactivity for APs with DAB substrate (brown color), the sections were then incubated with antibodies for GMF or iNOS or NF-κB at 4°C for overnight. The second immunoperoxidase reaction was developed with Vector SG substrate (blue color). GMF, iNOS and NF-κB (blue color, arrows) were co-localized at the vicinity of 6E10 labeled APs (brown color) in the AD brains (magnification = 200×). Bar graph show positive cells in these images.

Next, we studied if the upregulation of GMF, iNOS or NF-κB p65 are associated with APs in AD brains. We first imunostained for APs with 6E10 antibody followed by staining for GMF or iNOS or NF-κB (Figure 5). Our results show co-localization of APs (brown color, arrowheads) with GMF (blue color, arrows) or iNOS (arrows) or NF-κB (arrows) at the vicinity of APs (brown color) in the parahippocampal gyrus of AD brains (Figure 5). Our results indicate that GMF, iNOS and NF-κB are closely associated with each other in the pathogenesis of AD. Positive cells were counted from these images as shown in bar graph (Figure 5). GMF, iNOS and NF-κB positive cells were 39, 32 and 37, respectively.

### IHC of FASN, and Double Labeling of FASN with AT8, and FASN with 6E10

It has been shown that the mitochondrial UCPs such as the UCP2 is a regulator of energy homeostasis through *de novo* lipid synthesis, which involves the enzyme FASN for lipogenesis. In a previous study (Moon et al., 2015), it was demonstrated that UCP2 regulates the inflammatory responses mediated by NLRP3 inflammasome of cells by increase in FASN activity. Also, a recent review (Olsen and Singhrao, 2016) discussed the role of NLRP3 in AD pathogenesis. In our ongoing studies (unpublished observation) we have seen that down-regulation of GMF abrogates NLRP3 activation.

**Figure 6:**
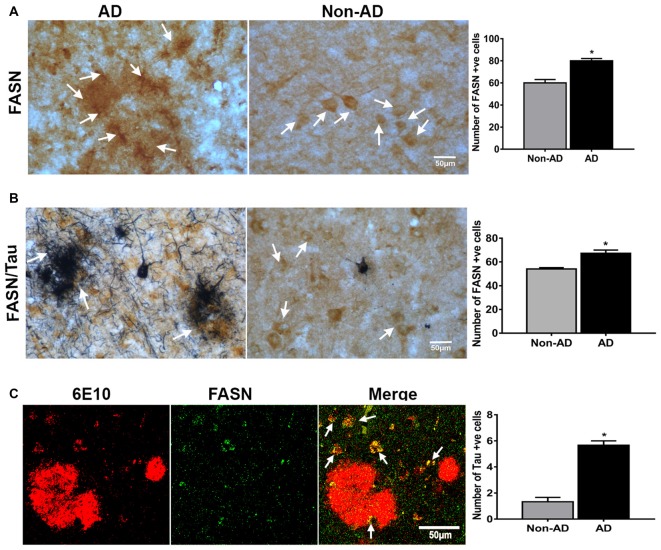
**IHC of fatty acid synthase (FASN), and double labeling of FASN with AT8, and FASN with 6E10. (A)** Immunohistochemical staining of FASN in AD and non-AD brain sections. Note the presence of strong immunoreactive cells (arrows) in the plaque area. **(B)** Double immunostaining for FASN (DAB, brown color) and tau (AT8 antibody; blue-gray color, SG Vector) showing the association of these (magnification = 400×). Positive cells were counted from these images and presented in graphs. **(C)** Confocal microscopy double immunofluorescence labeling of 6E10 (red) and FASN (green) in the AD brain showing the colocalization (yellow color; white arrows) in the merged image (magnification 63× oil immersion objective). *Significantly increased (*p* < 0.05) as compared with Non-AD.

To understand the relevance of UCPs in relation to inflammatory mediators in plaque regions of AD; we conducted the single immunostaining of FASN in the AD and non-AD brain (Figure 6A). FASN immunoreacitvity was observed in the plaque area of AD brain. FASN was detected in neurons in the brain. Double immunohistochemical staining of FASN and tau in the AD brain showed tau labeled NFTs and FASN immunoreactive pyramidal neurons (Figure 6B). Positive cells were counted from these images and shown in bar graphs. Double immunofluorescence labeling for FASN and 6E10 showed co-localization of this enzyme and plaques (Figure 6C).

## Discussion

Previous studies have shown that entorhinal cortex of parahippocampal gyrus is affected from the early stages of AD pathogenesis (Braak and Braak, 1991, 1995; Gómez-Isla et al., 1996). Several imaging studies have shown atrophy in the entorhinal cortex and hippocampus in the early stages of AD that was responsible for the confusion and other memory related disorders in AD (deToledo-Morrell et al., 2007; Devanand et al., 2007; Villain et al., 2008). Due to normal aging processes and in the presence of AD, the parahippocampal gyrus is highly affected region of the brain and thus we have analyzed this area in the present study. The present study demonstrates down-regulation of UCP2 and UCP4 expression and upregulation of GMF in the parahippocampal gyrus of AD brains. We have localized the expression of GMF and UCP2 or UCP4 in the close vicinity of APs. We found that GMF expression is associated with up-regulation of iNOS as well as NF-κB p65 in AD brains. AD is also considered as a systemic disease with mitochondrial abnormalities. Mitochondrial dysfunction has been suggested to precede both CNS neuropathological symptoms and contributes to the peripheral metabolic abnormalities frequently observed in AD (Morris et al., 2014). Mitochondrial dysfunctions and upregulation of GMF have been previously reported in neurodegenerative diseases (Ho et al., 2012a; Thangavel et al., 2013). Neuronal cells are known to express mitochondrial UCP2, UCP3 and UCP4 (Mattson and Liu, 2003), and here we have studied UCPs in AD brains. We have previously reported GMF upregulation in several brain regions in AD patients in close association with plaques (Zaheer et al., 2011; Thangavel et al., 2012, 2013; Stolmeier et al., 2013). Further, we have also found that GMF overexpression in the glial cells was associated with upregulation of mitogen-activated protein kinase (MAPKs) and NF-kB *in vitro* (Zaheer et al., 2007b). Oxidative stress-induced cell damage by mitochondrial dysfunction is found in neurodegenerative diseases including AD and is mediated by ROS (Bonda et al., 2014; Persson et al., 2014). It has been shown (de la Monte and Wands, 2006) that oxidative stress mediated mitochondrial dysfunction occurs early in AD and progresses with severity. UCP2 and UCP4 are known to protect neurons from mitochondrial dysfunction, oxidative damage, cell survival, preserving ATP synthesis and implicated in neurodegenerative diseases such as in AD and PD (Ho et al., 2012b). Overexpression of UCP2 provides protection from oxidative stress in several cells including neuronal cells, cardio myocytes, macrophages and monocytes. Overexpression of UCP4 was also shown to suppress apoptosis and reduce oxidative stress (Ho et al., 2012b). Though a number of studies have shown that UCP2 expression protects cells from oxidative stress mediated injuries, induction of UCP2 expression may be a double-edged sword as it could also induce mitochondrial dysfunction and necrotic death (Mills et al., 2002; Sriram et al., 2002). Our studies confirm the results of the former study that GMF is upregulated in the AD brains (Thangavel et al., 2013), and this could be associated with decreased UCP2 expression.

GMF is expressed in various human brain regions (Zaheer et al., 2011; Thangavel et al., 2012) and in rat brains as have been previously reported using various IHC and immunofluorescence staining procedures (Lim et al., 1987; Wang et al., 1992). In the present study, we have observed an increase in the number of reactive astrocytes, and reduced UCP2 as well as UCP4 positive cells in AD brains when compared to non-AD brains by immunostaining. Decreased UCP2 and UCP4 levels may precipitate neuronal death in AD. Our double immunofluorescence staining results show the upregulation of GMF-positive cells, and down regulation of UCP2 and UCP4-positive cells in AD brains. Both increased GMF and decreased UCPs may increase the glial activation and neuronal death in AD. The presence of APs and NFTs were observed in the AD brains by thioflavin-S staining and 6E10 antibody in the present study. However, there is no significant association of UCPs with APs or NFTs that were observed in this study. UCPs may be quickly upregulated due to acute tissue insult in order to protect neurons form injury, as shown for ischemic lesions in the brain (Nakase et al., 2007).

As various inflammatory molecules are increased in AD brains, we have analyzed the expression of iNOS in AD brains in relation to GMF expression. Our findings suggest that iNOS expression is more in the close vicinity of 6E10 labeled APs. Interestingly, it has been reported that UCP2 expression in macrophage significantly reduced the ROS and iNOS gene expression after LPS treatment (Kizaki et al., 2002). TNF-α which is known to be elevated in AD; reduces expression of UCPs but induced iNOS expression through NO-dependent pathways (Merial et al., 2000). The increased iNOS expression in the present study may correlate with the decreased level of UCPs in AD brains. Previous studies have shown high levels of NOS in the astrocytes that are present around the APs (Wallace et al., 1997). We have observed in the present study that iNOS upregulation is co-localized with increased GMF in AD brains. Similarly, we also co-localized the expression of GMF and NF-κB in the cytoplasm of AD brains (Hunot et al., 1997). This increased activation of NF-κB at the vicinity of GMF upregulation suggest that GMF may be partly involved in the increased activation of NF-κB in the AD brains. NF-κB complexes can specifically control the transcriptional activity, inducing either harmful or beneficial effects depending upon the combination of NF-κB dimers. Previous study has shown that UCP4 expression is significantly regulated through the activation and inhibition of NF-κB signaling by the NF-κB-response element binding site within the promoter region of UCP4 (Ho et al., 2012a). Aβ in close vicinity of APs activates NF-κB in neurons and glia in AD (Kaltschmidt et al., 1997). The aberrant NF-κB activation could be due to increased Aβ, GMF and other proinflammatory factors in AD brains. The current results suggest that GMF-positive glial and neuronal cells surround APs, and that the glial cells show increased NF-κB in AD brains. Proinflammatory signals/molecules such as LPS down-regulate UCP2 through c-Jun N-terminal kinase (JNK) and p38 MAPK pathways. UCP2 down-regulation was shown to increase mitochondrial ROS production to promote MAPK activation (Emre et al., 2007), which is shown to be activated in AD. NO and ROS are two well-known pro-apoptotic mediators. GMF could activate MAPKs and NF-κB that leads to the release of several proinflammatory cytokines and chemokines to augment and sustain AD pathogenesis. Our present findings are in agreement with the previous findings that increased activation of NF-κB has been reported in the vicinity of APs in AD brains. The mechanism of down-regulation of UCPs in AD in relation to GMF needs further studies. We found that increased NF-κB expression is co-localized with up-regulated GMF levels in the parahippocampal gyrus in AD brains. Acute activation of NF-κB may be neuroprotective but prolonged chronic activation could lead to neurodegeneration in neurodegenerative diseases (Kaltschmidt et al., 1997). In conclusion, UCP2 and UCP4 are down regulated in AD brains with up-regulation of GMF expression in the glial cells along with increased iNOS and NF-κB activities thereby indicating that GMF plays a proinflammatory role in the pathogenesis of AD probably by promoting mitochondrial dysfunction through down regulation of the UCPs.

The association of mitochondrial UCP2 and UCP4 near plaques in AD brains provides the physiological relevance of these proteins in regulating the inflammatory processes in AD. It is our understanding that neuronal death in AD is an interplay of oxidative stress mediated events activating a cell death pathway such as brought about by the inflammasome complex leading to mitochondrial dysfunction. The role of the mitochondrial UCPs is to regulate the cellular bioenergetics by its action on the lipid synthesis catalyzed by FASN through intermediates from the tricarboxylic acid cycle. Our ongoing work positions GMF as a regulator of the NLRP3 inflammasome complex.

From our current observations, we propose that mitochondrial dynamics in disease conditions is regulated on the one hand by UCPs through their action on FASN and on the other hand by GMF through its action on the NLRP3 inflammasome. In this context, earlier study (Iyer et al., 2013) has shown that NLRP3 binds to and is activated by the mitochondrial phospholipid cardiolipin. Future studies will delineate the exact mechanism of regulation of UCPs expression and activation by GMF using animal models of AD.

## Author Contributions

All authors contributed to the conception or design of the work; or analysis, or interpretation of data for the work.

## Conflict of Interest Statement

The authors declare that the research was conducted in the absence of any commercial or financial relationships that could be construed as a potential conflict of interest.

## References

[B1] Bevilaqua L. R. Rossato J. I. Bonini J. S. Myskiw J. C. Clarke J. R. Monteiro S.. (2008). The role of the entorhinal cortex in extinction: influences of aging. Neural Plast. 2008:595282. 10.1155/2008/595282 18584042 PMC2435227

[B2] Bonda D. J. Wang X. Lee H. G. Smith M. A. Perry G. Zhu X. (2014). Neuronal failure in Alzheimer’s disease: a view through the oxidative stress looking-glass. Neurosci. Bull. 30, 243–252. 10.1007/s12264-013-1424-x 24733654 PMC4097013

[B3] Braak H. Braak E. (1991). Neuropathological stageing of Alzheimer-related changes. Acta Neuropathol. 82, 239–259. 10.1007/bf00308809 1759558

[B4] Braak H. Braak E. (1995). Staging of Alzheimer’s disease-related neurofibrillary changes. Neurobiol. Aging 16, 271–278; discussion 278–284. 10.1016/0197-4580(95)00021-6 7566337

[B5] Conti B. Sugama S. Lucero J. Winsky-Sommerer R. Wirz S. A. Maher P.. (2005). Uncoupling protein 2 protects dopaminergic neurons from acute 1,2,3,6-methyl-phenyl-tetrahydropyridine toxicity. J. Neurochem. 93, 493–501. 10.1111/j.1471-4159.2005.03052.x 15816872

[B6] de la Monte S. M. Wands J. R. (2006). Molecular indices of oxidative stress and mitochondrial dysfunction occur early and often progress with severity of Alzheimer’s disease. J. Alzheimers Dis. 9, 167–181. 10.3233/jad-2006-9209 16873964

[B7] deToledo-Morrell L. Stoub T. R. Wang C. (2007). Hippocampal atrophy and disconnection in incipient and mild Alzheimer’s disease. Prog. Brain Res. 163, 741–753. 10.1016/s0079-6123(07)63040-4 17765748

[B8] Devanand D. P. Pradhaban G. Liu X. Khandji A. De Santi S. Segal S.. (2007). Hippocampal and entorhinal atrophy in mild cognitive impairment: prediction of Alzheimer disease. Neurology 68, 828–836. 10.1212/01.WNL.0000256697.20968.d7 17353470

[B9] Emre Y. Hurtaud C. Nübel T. Criscuolo F. Ricquier D. Cassard-Doulcier A. M. (2007). Mitochondria contribute to LPS-induced MAPK activation via uncoupling protein UCP2 in macrophages. Biochem. J. 402, 271–278. 10.1042/bj20061430 17073824 PMC1798432

[B10] Gómez-Isla T. Price J. L. McKeel D. W. Jr. Morris J. C. Growdon J. H. Hyman B. T. (1996). Profound loss of layer II entorhinal cortex neurons occurs in very mild Alzheimer’s disease. J. Neurosci. 16, 4491–4500. 8699259 10.1523/JNEUROSCI.16-14-04491.1996 PMC6578866

[B11] Granic I. Dolga A. M. Nijholt I. M. van Dijk G. Eisel U. L. (2009). Inflammation and NF-κB in Alzheimer’s disease and diabetes. J. Alzheimers Dis. 16, 809–821. 10.3233/JAD-2009-0976 19387114

[B12] Ho J. W. Ho P. W. Liu H. F. So D. H. Chan K. H. Tse Z. H.. (2012a). UCP4 is a target effector of the NF-κB c-Rel prosurvival pathway against oxidative stress. Free Radic. Biol. Med. 53, 383–394. 10.1016/j.freeradbiomed.2012.05.002 22580300

[B13] Ho P. W. Ho J. W. Liu H. F. So D. H. Tse Z. H. Chan K. H.. (2012b). Mitochondrial neuronal uncoupling proteins: a target for potential disease-modification in Parkinson’s disease. Transl. Neurodegener. 1:3. 10.1186/2047-9158-1-3 23210978 PMC3506996

[B14] Hof P. R. Bussière T. Gold G. Kövari E. Giannakopoulos P. Bouras C.. (2003). Stereologic evidence for persistence of viable neurons in layer II of the entorhinal cortex and the CA1 field in Alzheimer disease. J. Neuropathol. Exp. Neurol. 62, 55–67. 10.1093/jnen/62.1.55 12528818

[B15] Hunot S. Brugg B. Ricard D. Michel P. P. Muriel M. P. Ruberg M.. (1997). Nuclear translocation of NF-κB is increased in dopaminergic neurons of patients with parkinson disease. Proc. Natl. Acad. Sci. U S A 94, 7531–7536. 10.1073/pnas.94.14.7531 9207126 PMC23856

[B16] Iyer S. S. He Q. Janczy J. R. Elliott E. I. Zhong Z. Olivier A. K.. (2013). Mitochondrial cardiolipin is required for Nlrp3 inflammasome activation. Immunity 39, 311–323. 10.1016/j.immuni.2013.08.001 23954133 PMC3779285

[B17] Kaltschmidt B. Uherek M. Volk B. Baeuerle P. A. Kaltschmidt C. (1997). Transcription factor NF-κB is activated in primary neurons by amyloid β peptides and in neurons surrounding early plaques from patients with Alzheimer disease. Proc. Natl. Acad. Sci. U S A 94, 2642–2647. 10.1073/pnas.94.6.2642 9122249 PMC20142

[B18] Kamat P. K. Kalani A. Kyles P. Tyagi S. C. Tyagi N. (2014). Autophagy of mitochondria: a promising therapeutic target for neurodegenerative disease. Cell Biochem. Biophys. 70, 707–719. 10.1007/s12013-014-0006-5 24807843 PMC4184921

[B19] Kaplan R. Zaheer A. Jaye M. Lim R. (1991). Molecular cloning and expression of biologically active human glia maturation factor-β. J. Neurochem. 57, 483–490. 10.1111/j.1471-4159.1991.tb03777.x 1712830

[B20] Kizaki T. Suzuki K. Hitomi Y. Taniguchi N. Saitoh D. Watanabe K.. (2002). Uncoupling protein 2 plays an important role in nitric oxide production of lipopolysaccharide-stimulated macrophages. Proc. Natl. Acad. Sci. U S A 99, 9392–9397. 10.1073/pnas.142206299 12089332 PMC123151

[B21] Lim R. Hicklin D. J. Miller J. F. Williams T. H. Crabtree J. B. (1987). Distribution of immunoreactive glia maturation factor-like molecule in organs and tissues. Brain Res. 430, 93–100. 10.1016/0165-3806(87)90179-9 3297258

[B22] Lim R. Miller J. F. Zaheer A. (1989). Purification and characterization of glia maturation factor beta: a growth regulator for neurons and glia. Proc. Natl. Acad. Sci. U S A 86, 3901–3905. 10.1073/pnas.86.10.3901 2726756 PMC287249

[B23] Lim R. Zaheer A. Lane W. S. (1990). Complete amino acid sequence of bovine glia maturation factor β. Proc. Natl. Acad. Sci. U S A 87, 5233–5237. 10.1073/pnas.87.14.5233 2196564 PMC54297

[B24] Litvinova L. Atochin D. N. Fattakhov N. Vasilenko M. Zatolokin P. Kirienkova E. (2015). Nitric oxide and mitochondria in metabolic syndrome. Front. Physiol. 6:20. 10.3389/fphys.2015.00020 25741283 PMC4330700

[B25] Lu M. Sun X. L. Qiao C. Liu Y. Ding J. H. Hu G. (2014). Uncoupling protein 2 deficiency aggravates astrocytic endoplasmic reticulum stress and nod-like receptor protein 3 inflammasome activation. Neurobiol. Aging 35, 421–430. 10.1016/j.neurobiolaging.2013.08.015 24041971

[B26] Mattson M. P. Liu D. (2003). Mitochondrial potassium channels and uncoupling proteins in synaptic plasticity and neuronal cell death. Biochem. Biophys. Res. Commun. 304, 539–549. 10.1016/s0006-291x(03)00627-2 12729589

[B27] Merial C. Bouloumie A. Trocheris V. Lafontan M. Galitzky J. (2000). Nitric oxide-dependent downregulation of adipocyte UCP-2 expression by tumor necrosis factor-α. Am. J. Physiol. Cell Physiol. 279, C1100–C1106. 11003590 10.1152/ajpcell.2000.279.4.C1100

[B28] Mills E. M. Xu D. Fergusson M. M. Combs C. A. Xu Y. Finkel T. (2002). Regulation of cellular oncosis by uncoupling protein 2. J. Biol. Chem. 277, 27385–27392. 10.1074/jbc.M111860200 12011039

[B29] Moon J. S. Lee S. Park M. A. Siempos I. I. Haslip M. Lee P. J.. (2015). UCP2-induced fatty acid synthase promotes NLRP3 inflammasome activation during sepsis. J. Clin. Invest. 125, 665–680. 10.1172/JCI78253 25574840 PMC4319445

[B30] Morris J. K. Honea R. A. Vidoni E. D. Swerdlow R. H. Burns J. M. (2014). Is Alzheimer’s disease a systemic disease? Biochim. Biophys. Acta 1842, 1340–1349. 10.1016/j.bbadis.2014.04.012 24747741 PMC4126236

[B31] Nakase T. Yoshida Y. Nagata K. (2007). Amplified expression of uncoupling proteins in human brain ischemic lesions. Neuropathology 27, 442–447. 10.1111/j.1440-1789.2007.00815.x 18018477

[B32] Olsen I. Singhrao S. K. (2016). Inflammasome involvement in Alzheimer’s disease. J. Alzheimers Dis. 54, 45–53. 10.3233/JAD-160197 27314526

[B33] Pekny M. Nilsson M. (2005). Astrocyte activation and reactive gliosis. Glia 50, 427–434. 10.1002/glia.20207 15846805

[B34] Persson T. Popescu B. O. Cedazo-Minguez A. (2014). Oxidative stress in Alzheimer’s disease: why did antioxidant therapy fail? Oxid. Med. Cell. Longev. 2014:427318. 10.1155/2014/427318 24669288 PMC3941783

[B35] Porchet R. Probst A. Bouras C. Dráberová E. Dráber P. Riederer B. M. (2003). Analysis of glial acidic fibrillary protein in the human entorhinal cortex during aging and in Alzheimer’s disease. Proteomics 3, 1476–1485. 10.1002/pmic.200300456 12923773

[B36] Reddy P. H. Reddy T. P. (2011). Mitochondria as a therapeutic target for aging and neurodegenerative diseases. Curr. Alzheimer Res. 8, 393–409. 10.2174/156720511795745401 21470101 PMC3295247

[B37] Rose G. Crocco P. De Rango F. Montesanto A. Passarino G. (2011). Further support to the uncoupling-to-survive theory: the genetic variation of human UCP genes is associated with longevity. PLoS One 6:e29650. 10.1371/journal.pone.0029650 22216339 PMC3246500

[B38] Sriram K. Benkovic S. A. Miller D. B. O’Callaghan J. P. (2002). Obesity exacerbates chemically induced neurodegeneration. Neuroscience 115, 1335–1346. 10.1016/s0306-4522(02)00306-8 12453501

[B39] Stolmeier D. Thangavel R. Anantharam P. Khan M. M. Kempuraj D. Zaheer A. (2013). Glia maturation factor expression in hippocampus of human Alzheimer’s disease. Neurochem. Res. 38, 1580–1589. 10.1007/s11064-013-1059-3 23640177 PMC3692596

[B40] Streit W. J. Braak H. Xue Q. S. Bechmann I. (2009). Dystrophic (senescent) rather than activated microglial cells are associated with tau pathology and likely precede neurodegeneration in Alzheimer’s disease. Acta Neuropathol. 118, 475–485. 10.1007/s00401-009-0556-6 19513731 PMC2737117

[B41] Thangavel R. Kempuraj D. Stolmeier D. Anantharam P. Khan M. Zaheer A. (2013). Glia maturation factor expression in entorhinal cortex of Alzheimer’s disease brain. Neurochem. Res. 38, 1777–1784. 10.1007/s11064-013-1080-6 23715664 PMC3735652

[B42] Thangavel R. Stolmeier D. Yang X. Anantharam P. Zaheer A. (2012). Expression of glia maturation factor in neuropathological lesions of Alzheimer’s disease. Neuropathol. Appl. Neurobiol. 38, 572–581. 10.1111/j.1365-2990.2011.01232.x 22035352 PMC3290752

[B43] Van Hoesen G. W. Augustinack J. C. Dierking J. Redman S. J. Thangavel R. (2000). The parahippocampal gyrus in Alzheimer’s disease: clinical and preclinical neuroanatomical correlates. Ann. N Y Acad. Sci. 911, 254–274. 10.1111/j.1749-6632.2000.tb06731.x 10911879

[B44] Villain N. Desgranges B. Viader F. de la Sayette V. Mézenge F. Landeau B.. (2008). Relationships between hippocampal atrophy, white matter disruption, and gray matter hypometabolism in Alzheimer’s disease. J. Neurosci. 28, 6174–6181. 10.1523/JNEUROSCI.1392-08.2008 18550759 PMC2902815

[B45] Viña J. Lloret A. Vallés S. L. Borrás C. Badía M. C. Pallardó F. V.. (2007). Mitochondrial oxidant signalling in Alzheimer’s disease. J. Alzheimers Dis. 11, 175–181. 10.3233/jad-2007-11205 17522442

[B46] Wallace M. N. Geddes J. G. Farquhar D. A. Masson M. R. (1997). Nitric oxide synthase in reactive astrocytes adjacent to β-amyloid plaques. Exp. Neurol. 144, 266–272. 10.1006/exnr.1996.6373 9168828

[B47] Wang B. R. Zaheer A. Lim R. (1992). Polyclonal antibody localizes glia maturation factor β-like immunoreactivity in neurons and glia. Brain Res. 591, 1–7. 10.1016/0006-8993(92)90971-b 1446220

[B48] Wong A. Lüth H. J. Deuther-Conrad W. Dukic-Stefanovic S. Gasic-Milenkovic J. Arendt T.. (2001). Advanced glycation endproducts co-localize with inducible nitric oxide synthase in Alzheimer’s disease. Brain Res. 920, 32–40. 10.1016/s0006-8993(01)02872-4 11716809

[B49] Xiong Z. Thangavel R. Kempuraj D. Yang E. Zaheer S. Zaheer A. (2014). Alzheimer’s disease: evidence for the expression of interleukin-33 and its receptor ST2 in the brain. J. Alzheimers Dis. 40, 297–308. 10.3233/JAD-132081 24413615 PMC4015800

[B50] Zaheer A. Sahu S. K. Wu Y. Zaheer A. Haas J. Lee K.. (2007a). Diminished cytokine and chemokine expression in the central nervous system of GMF-deficient mice with experimental autoimmune encephalomyelitis. Brain Res. 1144, 239–247. 10.1016/j.brainres.2007.01.075 17316572 PMC1899479

[B51] Zaheer A. Zaheer S. Sahu S. K. Knight S. Khosravi H. Mathur S. N.. (2007b). A novel role of glia maturation factor: induction of granulocyte-macrophage colony-stimulating factor and pro-inflammatory cytokines. J. Neurochem. 101, 364–376. 10.1111/j.1471-4159.2006.04385.x 17250654

[B53] Zaheer S. Thangavel R. Sahu S. K. Zaheer A. (2011). Augmented expression of glia maturation factor in Alzheimer’s disease. Neuroscience 194, 227–233. 10.1016/j.neuroscience.2011.07.069 21835226 PMC3183233

[B52] Zaheer A. Zaheer S. Thangavel R. Wu Y. Sahu S. K. Yang B. (2008). Glia maturation factor modulates β-amyloid-induced glial activation, inflammatory cytokine/chemokine production and neuronal damage. Brain Res. 1208, 192–203. 10.1016/j.brainres.2008.02.093 18395194 PMC2587299

